# A study of college students’ perceptions of utilizing automatic speech recognition technology to assist English oral proficiency

**DOI:** 10.3389/fpsyg.2022.1049139

**Published:** 2022-12-08

**Authors:** Jiaxin Liu, Xianghu Liu, Chuan Yang

**Affiliations:** College of Foreign Languages, Bohai University, Jinzhou, China

**Keywords:** artificial intelligence, automatic speech recognition technology, IELTS speaking part 3, oral English training, human-computer interaction, student perceptions of ASR technology

## Abstract

For English as a Foreign Language (EFL) students, automatic speech recognition (ASR) technology is the most potential assistant tool to help them improve their spoken English ability. The primary purpose of this study is to investigate learners’ perceptions towards ASR technology after it is applied to traditional classrooms. This study selected 249 English majors from a university in Northeastern China as samples and divided them into a control group consisting of 124 students and an experimental group including 125 students. The participants of two groups used ASR technology in the process of oral practice, and the experimental group also added teacher’s guidance compared with the control group. The teacher gives more detailed instruction in speaking based on the scores provided by ASR technology. Participants needed to complete relevant questionnaires and learning reflective journals during the experiment. The results of the study showed that both participants and instructors held positive and satisfactory attitudes towards the potential of ASR in oral training and believed that the technology could meet many of their needs such as the scoring system to help them more intuitively understand the real speaking level. The findings of this paper will give some implications to oral English teaching in the future.

## Introduction

Speaking has always been the most challenging English skill for students who learn English as a foreign language. Some studies show that English language learners face many problems when communicating in English such as lack of oral practice opportunities (Wang and Young, 2014; Ahn and Lee, 2016), anxiety during oral expression (Coskun, 2016), and lack of confidence and motivation (Al-Sobhi and Preece, 2018). In recent years, the developments in mobile devices and educational applications provided numerous learning resources for mobile assisted language learning (MALL). MALL refers to second language learning or foreign language learning by using different mobile devices such as mobile phones, tablets and personal digital assistants (PDAs) (Stockwell, 2022). It primarily studies the application of mobile technology in language learning (Rahimi and Miri, 2014). Compared with the traditional teaching, learners do not need to sit in the classroom to acquire knowledge. MALL is a freer learning way that learners can choose to learn a second language at anytime and anywhere (Egbert et al., 2012; Gafni et al., 2017). Therefore, MALL can be considered as an ideal way to address learning styles that are limited by time and places (Miangah and Nezarat, 2012), because it can consider not only technology and mobility of learning, but also the mobility of learners (Hashim et al., 2017; Pegrum, 2019; Arvanitis and Krystalli, 2021). In the field of MALL, researchers are mostly concerned about how to apply theory to practice and apply technology to teaching to help teachers better carry out teaching practices (Zou and Yan, 2014; Zou et al., 2018; Stockwell, 2022). Existing research results indicated that MALL is an effective mobile language learning tool and it can enhance learners’ language skills such as speaking and writing, communication skills, learning motivation and confidence (Kondo et al., 2012; Golonka et al., 2014; Toland et al., 2016; Mortazavi et al., 2021; Stockwell, 2022). For example, Gonulal (2019) focused on exploring the utility of MALL-based applications for students’ language learning and found that MALL was able to improve learners’ overall language skills, especially communication skills.

Automatic speech recognition (ASR) technology is a promising educational technology in MALL, which can provide a convenient way for learners to learn languages and practice pronunciation. It is usually used as an identification system embedded in mobile devices or educational applications. Also, ASR technology is principal to evaluate learners’ conversations accurately and objectively in English teaching because it can help teachers reduce their workload. If ASR technology is integrated into traditional phonetic teaching, an ASR scoring system ought to be developed to be virtually consistent with human evaluation and judgment of samples (Neri et al., 2008) to ensure the accuracy of scoring. At present, applications based on ASR on the market are relatively mature, but if these are to be applied in the classroom, some changes should be made to the corresponding evaluation system, such as adjusting the scoring weight (Liu et al., 2019).

Accurate and timely feedback is a significant part in English teaching and learning (Rogerson-Revell, 2021). In recent years, many English teaching applications equipped with ASR technology on the market aims to help learners better perceive their pronunciation problems and allow them to effectively train themselves. There are many applications based on ASR technology on the market, each with a different form of voice feedback (Evers and Chen, 2020; Rogerson-Revell, 2021). Other applications are presented to the user in the form of diagrams such as audio waveforms, in which users can observe the tone contour of their own speech, which can improve the perception of intonation through the image (Olson, 2014). This method is more suitable for phonetics teachers’ guidance and explanation because most learners do not have a deep understanding of phonetic knowledge and cannot accurately extract useful information from diagrams. Some ASR technologies provide feedback in the form of verbal descriptions and articulation charts. This feedback can improve learners’ pronunciation gradually and enhance the accuracy of segmentation (Bogach et al., 2021).

Additionally, leaner autonomy plays an important part in language learning and teaching. Little (1991) believed that if learners can master learner autonomy, they will be more focused in the learning process, thus improving the effectiveness of learning. It can be seen from previous studies that ASR technology can play a crucial role in promoting learner autonomy. Kruk (2012) found that the use of digital technology and online resources could promote learner autonomy and pronunciation teaching. After using mobile applications based on ASR technology for pronunciation learning, Cavus (2016) emphasized that e-learning could give learners more learning motivation than traditional learning. Similarly, Mccrocklin (2016) findings showed that the experimental group with ASR technology had a significant increase in autonomous beliefs, and the feedback provided by ASR can enable learners to practice independently. All these research results indicated that ASR technology can improve learner autonomy.

Furthermore, ASR technology has been proven to be effective for learners’ pronunciation in language teaching and learning. In order to explore the effectiveness of feedback from ASR, Mroz (2018) research findings demonstrated that the use of software based on the ASR system could improve learners’ pronunciation and gradually enhance their speaking ability. The potential and advantages of electronic technology are widely recognized (Sidgi and Shaari, 2017; Kholis, 2021). For example, Pourhosein Gilakjani and Rahimy (2020) believed that the use of electronic technology in traditional pronunciation classes brings positive effects to learners. Liakin et al. (2015) also applied ASR technology to traditional phonetic teaching, and the research results showed that ASR technology could have a positive impact on teaching effects. Xodabande (2017) investigated whether new technologies could promote English pronunciation teaching and found that ASR technology could play a significant role in improving oral English pronunciation skills. The results of their study confirmed this hypothesis, finding that CAPT-based (Computer Assisted Pronunciation Teaching) classroom instruction significantly improved students’ speaking ability. Evers and Chen (2020) examined the differences of two training methods (peer feedback and individual practice) by using ASR systems in Taiwan. The results showed that peer feedback was more effective in correcting pronunciation and that learners in the experimental group were more satisfied with the software than those in the control group. From these experimental studies, in spite of imperfection of ASR technology, it has obviously played a vital and positive role in the field of education (Saito and Lyster, 2012; Lee et al., 2015), especially in improving pronunciation and oral ability (Golonka et al., 2014).

In traditional education, the guidance of teachers plays a significant role in teaching speaking. Kaendler et al. (2015) argued that learners’ academic progress is inseparable from the teaching decisions made by teachers. Meanwhile, during the learning process, teachers will monitor students as supervisors. When students encounter unsolved problems, teachers will offer help when necessary (Van de Pol et al., 2015). This study provided a teaching and self-directed learning model that combined traditional language learning plus teacher guidance with ASR technology as the core to help Chinese college students improve their oral English. The primary purpose of this study is to conduct further research according to the characteristics of ASR, to investigate student perceptions of the learning software with an ASR system because learners’ attitudes towards ASR technology affect their learning performance in the target language (Cheng and Chen, 2022). Specifically, this paper attempts to address the following two research questions:

1. What are the learners’ perceptions of using ASR system?
2. Is there a significant difference in the degree of satisfaction between those who do not receive teacher guidance and those who do receive teacher guidance?

## Methods and research design

### Research participants

A total of 249 participants with an average age of 19 years from a university in Northeastern China participated in this study. Among them, male and female students accounted for 7.58 and 92.42%, respectively. All students had nearly 11 years of experience in learning English. The choice of sampling in this study was to match with the research purpose, one of sampling principles in education research (Cohen et al., 2018). The participants were divided into two groups, an experimental group (EG) (124 students) taught by teacher A, who gave them guidance in terms of IETLS Speaking Part 3 in classroom teaching, and a control group (CG) (125 students) taught by teacher B, who gave no guidance about it. As the content of the English test in the college entrance examination excluded the oral English test, an oral English level test was conducted so that their oral English proficiency was known before the experiment. The speaking proficiency pre-test results showed that EG students scored 53 out of 100 points on average, while CG students scored 51.7. Both EG and CG added ASR technology to assist oral training in the process of learning IELTS speaking Part 3. But the difference was that EG added additional teacher guidance for the content of ASR feedback.

### Instruments

In this study, data were collected through questionnaires and reflective learning journals to obtain the real thoughts and perceptions of learners, and SPSS19.0 statistical software was used for data analysis.

#### The Brother IELTS App and the IELTS Fluent Speaking App

As the teaching content in class is related to the IELTS speaking Part-3 test, the following two software with ASR systems as assistant tools to learn oral English.

The Brother IELTS App is a software for learning spoken English. Learners can accumulate numerous spoken language materials from the software in the process of autonomous learning. Developers set up authentic IELTS speaking tests and simulation tests for learners to practice in this app as well as AI training function with ASR technology. This function is mainly designed for learners’ pronunciation. To be specific, this feature allows learners to read the sentences displayed on the screen or imitate native speakers’ intonation to be recorded. After the learner completes the recording, the system will automatically score and give feedback according to his or her voice, aiming to provide assistance with the oral pronunciation improvement.

Unlike the Brother IELTS software, the IELTS Fluent Speaking App installs an ASR system to assess a learner’s overall speaking ability. The participants were given a recorded speaking test to score in terms of fluency, grammar, pronunciation and vocabulary. They were asked to use the software’s capabilities after class to test their performance in daily speaking exercises. Learners can only feel the changes in their oral ability through the fluctuating scores given by the ASR technology. Similarly, due to the characteristics of this app, the IELTS Fluent Speaking App was also used as a testing tool.

#### Questionnaires

In the field of education, most investigators use the research method of questionnaire to collect data (Cohen et al., 2018). Compared with other data collection methods, questionnaires have many advantages, such as low cost, anonymity and reduction of biasing errors (Dalati and Marx Gómez, 2018). Since participants will not be interfered or influenced by others in the process of completing the questionnaire, the questionnaire is often easier to answer and can reduce the boredom and fatigue of respondents (Bryman, 2016). In order to fully understand the participants’ opinions and perceptions towards software equipped with an ASR system, participants were asked to fill out a questionnaire after the final speaking test. The questionnaire used in this study was divided into three parts. Part 1 was demographic information about the participants such as age and gender. Part 2 was designed according to the Likert scale, with a total of 26 questions, aimed at understanding the participants’ opinions on the two apps used in the process of independent learning. Part 3 consisted of four subjective questions, aiming to further understand the learners’ views on the relationship between teachers and technology, and their perceptions of the two learning apps. The validity and reliability of the scale in Part 2 were studied. The results showed that Cronbach’s Alpha value was 0.971, greater than 0.9, which meant that the reliability quality of the questionnaire was very high (Eisinga et al., 2013). There are three reasons for choosing to use the same questionnaire in the pre-experiment and post-experiment. Firstly, issuing the same questionnaire can reduce measurement error (Gehlbach and Brinkworth, 2011). Secondly, it can improve the reliability and validity of the questionnaire (Gehlbach and Brinkworth, 2011). Finally, researchers can observe changes in learners’ attitudes toward the use of software through the same questionnaire measured at different times (Bourdas and Zacharakis, 2020; Cicha et al., 2021).

#### Reflective learning journals

Because respondents’ answers to the questionnaire were limited, researchers may have missed some detailed, in-depth information. The characteristics of the learning journal are that the data collected are comprehensive and true, and cover more information about learners (Miller and Brewer, 2003). Compared with the questionnaire, the content recorded in the reflective journal was not limited by specific questions (Mackey and Gass, 2021), and based on the questionnaire, the reflective journal was used to further explore learners’ psychological activities. Compared to interviews, a reflective journal is a more flexible method of data collection that learners complete according to their personal time schedules (Mackey and Gass, 2021). Therefore, each learner needs to complete a reflective learning journal in order to obtain more reliable data.

After the experiment began, learners were required to write reflective journals weekly. The participants wrote journals based on the effectiveness of the oral training, the satisfaction of the software, and the teachers’ role in their oral training. Among them, the effectiveness of oral training is to understand whether learners can improve their oral expression ability through ASR software; The satisfaction of the software is to understand whether the learner is satisfied with the functions provided by the software. Learners can discuss the practicability, operability, convenience, content interest and ASR recognition accuracy of the software. The purpose of discussing the teachers’ role in oral training is to understand whether ASR technology can replace the teachers’ role in English teaching in order to analyze the advantages and disadvantages of the two learning approaches. In short, learners can have a clearer understanding of their own shortcomings through the combination of personal feelings and ASR feedback information and can have more targeted exercises in following week’s practice. Additionally, researchers can have a more comprehensive understanding of the changes in learners’ behavior, learning attitude and learning situation during the experiment to better arrange after-class learning activities.

### Research procedures

The experiment lasted for a semester. At the beginning of the experiment, all participants were introduced to the use of the learning software and given a questionnaire to fill out. During the experiment, the teacher combined the oral materials on the software and the original classroom teaching content to teach students, and assigned the oral topics related to the classroom content and made the students practice independently through the software. Compared with CG students, EG received the teacher’s instruction and guidance for their oral work after class weekly. The reason why teacher intervention is included in the experiment process is to help EG students better understand the meanings of the scores and feedback provided by the ASR system to enhance their oral English ability. Additionally, the researchers wanted to observe whether ASR can be brought into the traditional oral classroom and better assist teachers in oral training through teacher intervention in the future. At the end of the experiment, the two-group learners were asked to complete the questionnaire again. Researchers collected data from the two questionnaires for comparison and analysis.

### Data analysis

Qualitative and quantitative research methods were adopted in this study. Data were collected through 249 valid questionnaires and reflective learning journals. Data were analyzed by using SPSS 19.0 software and selecting independent samples t-test to compare the differences between EG and CG. Data of reflective journals were analyzed by the method of content analysis.

## Results

Questionnaire answers and learners’ reflective learning journals were analyzed so as to obtain results on participant perceptions of the two apps, namely the Brother IELTS App and the IELTS Fluent Speaking App. The questionnaire had 26 objective questions related to learning software technology for analysis such as user interface and operation experience.

Tables 1, 2 respectively show 12 opinions about the Brother IELTS App and 14 opinions about the IELTS Fluent Speaking App in the questionnaire survey, together with the average scores of EG and CG. The average scores represent how strongly participants agreed with the ideas. A higher average means a higher degree of agreement.

**Table 1:** The average score of the participants for the questionnaire about the Brother IELTS App.

Items	Mean	
	EG	CG
BIA is easy to use and browse	4.24	4.12
The user interface of BIA is attractive	4.08	3.91
The learning content of BIA is very rich	4.32	4.16
BIA provides a lot of oral materials	4.34	4.24
I often upload my oral audio to BIA	3.69	3.67
I often play other students’ audio in BIA and learn from it	3.92	3.8
The oral audio of other students in BIA has helped me a lot in my oral practice	4.16	3.95
BIA can help me improve my oral expression ability	4.32	4.18
The AI companion function of BIA provides pronunciation practice of different themes	4.27	4.12
The AI training partner function of BIA is very useful for improving pronunciation	4.24	4.15
If I study IELTS in the future, I will continue to use the BIA	4.3	4.11
I think the overall quality and sense of use of BIA is very good	4.27	4.1
Total	4.18	4.04

“BIA” represents “Brother IELTS App.”

**Table 2:** The average score of the participants for the questionnaire about the IELTS Fluent Speaking App.

Items	Mean	
	EG	CG
IFSA is easy to use and browse	4.19	4.1
The user interface of IFSA is very attractive	4.13	4.02
IFSA is very rich in content	4.32	4.06
The speech recognition mechanism in IFSA has high accuracy	4.21	4.07
The scoring function of IFSA can help me better understand my speaking level	4.32	4.14
The scoring function of IFSA will motivate me to practice speaking	4.33	4.21
The scoring results of IFSA are highly accurate	4.18	4.02
IFSA can help me improve my oral expression ability	4.38	4.13
Using IFSA to take the speaking test can relieve my tension	4.23	4.02
Using IFSA to take the speaking test can quickly let me know my score	4.33	4.18
I will use the scoring mechanism of IFSA for daily oral practice	4.28	4.07
Technical problems may occur during the use of IFSA. For example, the score is not visible for network reasons	3.76	3.74
I hope I can use testing software like IFSA for oral test in the future	4.27	4.09
I think the overall quality and usage of IFSA is very good	4.32	4.04
Total mean	4.23	4.06

“IFSA” represents “IELTS Fluent Speaking App.”

Table 1 indicates the participants’ feelings about the Brother IELTS App. Also, the two-group learners had a high sense of identity to the 12 viewpoints. The total average score of EG was 4.18, while the total average score of CG was 4.04, which is a 0.14 difference from the average score of EG. This shows that EG students had a higher sense of identity to learning software than CG.

Table 2 displays the participants’ attitudes towards the IELTS Fluent Speaking App. In summary, the two-group learners had a high sense of identity to the 14 viewpoints. Among them, the total average score of EG students was 4.23, while the total average score of CG was 4.06, or 0.17 lower than that of EG. This result shows that the two-group students had a positive attitude towards the test software. By contrast, EG students had a higher sense of identity to learning software than CG.

In order to better confirm whether there is a significant difference in the student perceptions in EG and CG towards software, the questionnaire data were analyzed by independent sample *t*-test with SPSS 19.0 statistical software.

Table 3 indicates that there is no significant difference between EG and CG students’ scores when faced with questions about the Brother IELTS App in the questionnaire (*df* = 22, *p* > 0.05, using SPSS 19.0 statistical software, similarly *hereinafter*). Although the scores of EG (*M* = 4.18, *SD* = 0.20) were higher than those of CG (*M* = 4.04, *SD* = 0.17), there were no significant differences, which means the two-group students had similar perceptions towards the Brother IELTS App.

**Table 3:** The statistical scales for the questionnaire about the two apps.

Questionnaire	EG		CG		*MD*	*t*
	*M*	*SD*	*M*	*SD*		
Brother IELTS App	4.18	0.20	4.04	0.17	0.14	1.82
IELTS Fluent Speaking App	4.23	0.15	4.06	0.11	0.17	3.34

Table 3 also shows that the scores obtained by EG and CG were significantly different (*df* = 26, *p* < 0.05) when answering questions about the IELTS Fluent Speaking App in the questionnaire. The average score of the two groups indicated that the score of EG (*M* = 4.23, *SD* = 0.15) was significantly higher than that of CG (*M* = 4.06, *SD* = 0.11), with a difference of 0.17. The results demonstrate that the two groups had positive perceptions towards the software, but the satisfaction of EG was significantly higher than that of CG.

The learner reflective journal data were consistent with the questionnaire findings. Most of the participants mentioned that learning software based on ASR technology had a significantly beneficial effect on their English-speaking learning. They commented positively on ASR, and believed that through visual score feedback, they were able to improve their English skills. However, some students asserted that the recognition function of ASR were affected by the environment and network of speakers, resulting in interruption of the use. As this was a bit of a hassle for them, they needed to find a quiet place with a good Internet signal before conducting the test. Regarding the ASR scoring function, students stated that the function showed their language levels they had simultaneously. Student 1 pointed out that the scoring function could help her know whether she had problems with her speaking fluency or pronunciation. Moreover, most of the learners are willing to add ASR technology to their oral practice in the future because ASR technology can generate learning motivation and promote learner autonomy. Student 2 emphasized this point: “In each practice, I can see my score rising, which lets me make a sense of achievement and full of motivation to practice my spoken English and look forward to getting a higher score next time.” Furthermore, some students put forward other advantages of ASR technology. As Student 3 noted, “I found the automatic grading function of the software very useful to me. I will not worry that my teacher will make unfair or incorrect assessment on my speaking level due to stereotyping or other reasons, because of my speaking level known in real time.” Student 4 agreed with the statement that using ASR in future speaking tests may increase the objectivity of assessment. These findings demonstrated that learners liked to use ASR technology to practice spoken English. Therefore, the application software based on ASR could enhance learners’ language skills and autonomous learning. ASR technology allows learners to practice speaking without time and locative limitation and obtain score feedback in real time. In summary, all participants had positive attitudes towards the scoring function of the ASR system and a high level of satisfaction with the ASR system.

**Figure 1:**
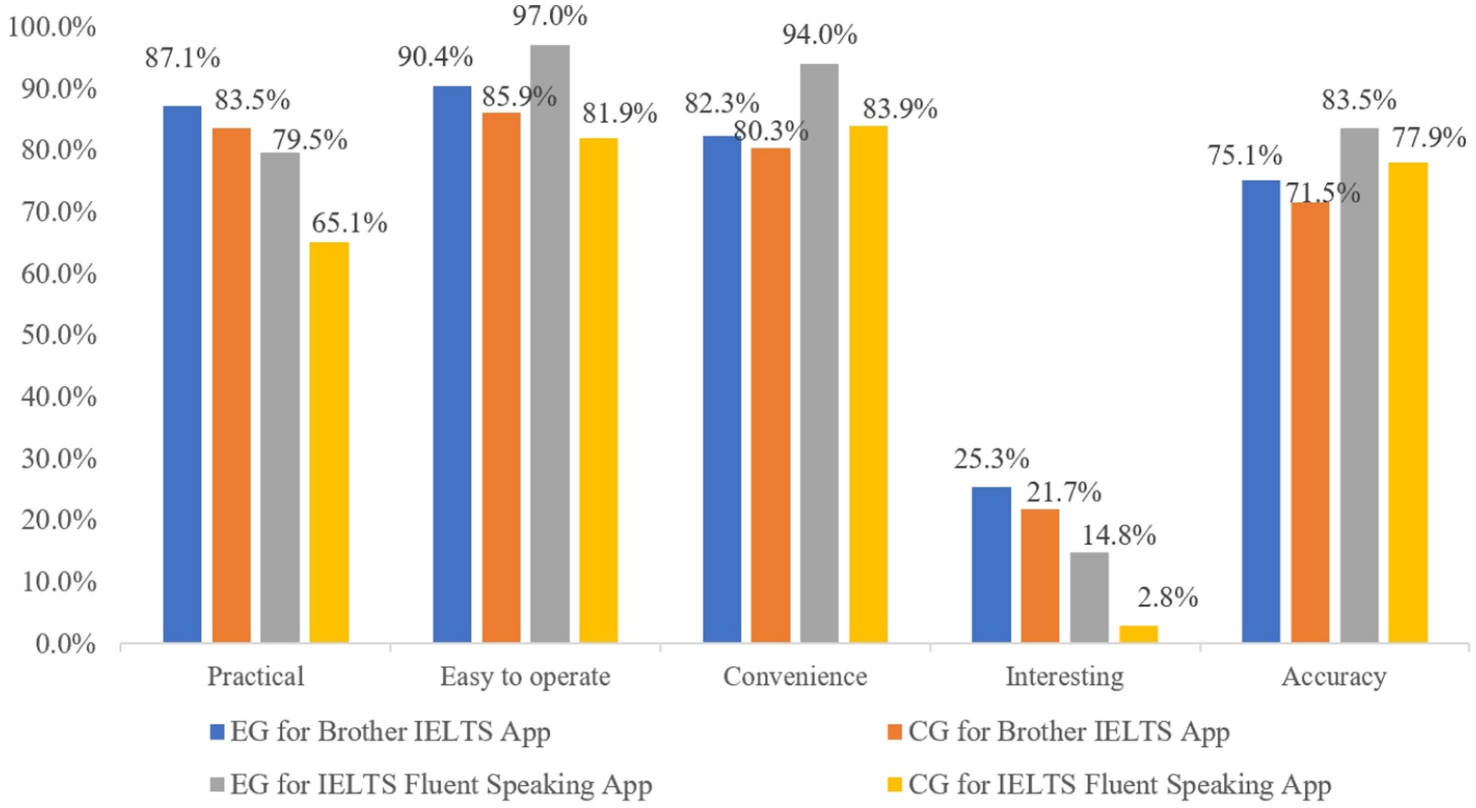
The distribution of EG and CG students’ statements about the two apps.

Figure 1 shows the distribution of the perceptions of the two groups towards the two apps from the five different aspects. The results show that learners are generally satisfied with both apps. For the Brother IELTS app, there was no significant difference in the satisfaction of EG and CG students on any factor, and the difference was within 5%. In the reflective journals, learners reported that the user-friendliness of the app saved them much learning time. For the IELTS Fluent Speaking App, EG and CG students had a large difference in satisfaction in all aspects, and the difference in satisfaction between the two groups was mostly more than 10%. When it came to accuracy, for example, learners had different opinions. Some students believed that the scores given by the grading system of the software deviated greatly from their actual level. Some students felt they could obtain an accurate assessment in quiet places. There are two possible reasons for the large difference in accuracy satisfaction between the two groups. The first one is that there are some differences during the oral test by using software, dependent upon quiet testing environments or headphones worn. The second reason could be that the recognition rate of the software’s ASR system needs to be improved. It is expected that the recognition rate of the software will be gradually improved in the future with software upgrades.

To summarize, the results of the two different analytical methods are consistent. The results indicated that the two-group learners had positive attitudes towards the two apps and held high evaluations. On the Brother IELTS app, both groups had similar levels of satisfaction with their use. However, EG were more satisfied with the IELTS Fluent Speaking App than CG students.

## Discussion

Two research questions in this paper are to investigate learners’ perceptions of learning software based on ASR system and explore whether different learning approaches will affect learners’ satisfaction. Next, two research questions will be discussed in detail.

### The students’ perceptions

Traditional oral training methods rely on teachers, which makes it difficult for learners to monitor their own pronunciation independently. However, the scoring function of ASR system can help them understand their oral level more clearly and carry out targeted practice to achieve the goals of improving their oral ability. Similarly, in the process of continuous practice, learners can observe their progress through the scores given by the ASR system, without blind training. Therefore, it is found in the questionnaire survey and reflective learning journals that the two software can not only help them improve their oral English ability, but also meet their needs in daily oral training. Additionally, the ease of using apps can potentially increase learners’ interest and motivation in oral English training and reduce the anxiety that arises when speaking English. More importantly, ASR systems will provide more objective scores than manual scores. In summary, the two-group learners had a positive attitude and a good opinion of the software based on the ASR system.

The results of this study were consistent with the findings of Pourhosein Gilakjani and Rahimy (2020) and Liakin et al. (2015) that participants had a positive attitude and satisfaction towards software based on ASR systems. Additionally, the results were similar to Mroz (2018) findings that ASR technology can improve learners’ English pronunciation skills. To summarize, software based on ASR technology is a very attractive learning tool for language learners, which can bring numerous positive effects to them.

### The differences in terms of satisfaction between EG and CG

In this study, the researchers used two software to investigate learners’ satisfaction with it. On the Brother IELTS app, the satisfaction of EG students was similar to that of CG, and there was no significant difference between the two. This was probably because in terms of training oral skills, the function of the Brother IELTS app focused more on the oral materials and the training of English pronunciation skills, which led to no significant satisfaction deviation between the two-group learners. In the IELTS Fluent Speaking App, the results changed. The results showed that there was a significant difference between the satisfaction of EG and CG students, and EG were significantly more satisfied with the software than CG. The reason for this change was likely to be that when students used ASR system to improve their oral English, for EG with teacher feedback, they could have a clearer understanding of specific language problems and a clear training plan with the teacher’s help. Although CG students can also know their language levels through ASR, it is challenging for them to practice deeply for a certain problem. Therefore, this requires the help and guidance from teachers.

This research result is similar to that of Evers and Chen (2020). There was a significant difference in satisfaction with the application among participants who adopted different learning strategies, whether there was a teacher feedback activity or not. Satisfaction was higher in EG than in CG.

## Conclusion

In this study, two learning applications based on ASR systems were investigated. Specifically, this study investigates learners’ perceptions of using the two software and explores whether learners will have different satisfaction due to different learning approaches. The research results suggested that most students believe that the two software with ASR system can be beneficial to the improvement of speaking ability, which are very useful and attractive learning software for them. Not only can the scoring function of the software enable learners to clearly see their oral English level and progress, but also the scoring mechanism can stimulate their intrinsic motivation, thus promoting them to continue to practice effectively. Although the recognition accuracy of ASR system was declined due to testing environments, most students held positive attitudes towards it. The results also indicated that different learning approaches can lead to different satisfaction of learners with the software. When using the IELTS Fluent Speaking App, the satisfaction of EG is higher than that of CG.

In previous studies, most teaching experiments related to ASR were tested in the form of *Read Aloud*, and the main purpose of researchers was to improve learners’ oral pronunciation through ASR systems. However, in this study, the researchers changed the type of ASR test in the past, and selected ASR software to improve the overall speaking ability of learners, not limited to a single spoken pronunciation. This study further explores the ASR technology based on previous studies, enriching the research results about the combination of ASR technology and language teaching. Furthermore, this paper introduces the ASR system with its recognition and feedback functions in detail and makes a critical analysis based on previous relevant literature to study more suitable learning tools for learners and put forward constructive suggestions for future teaching. Additionally, this study promotes the application of ASR system in oral English learning. So far, the research on improving oral English ability by ASR system is limited, especially for Chinese college students. Meanwhile, the sample size of this study is larger than that of previous experimental studies, which improves the accuracy of research results. As a result, ASR technology is used as an effective tool for oral evaluation in this study, which is a new attempt to reform the traditional assessment methods.

Although the current level of technology is unable to achieve human-machine free communication, it still has great advantages in the field of English teaching. For learners, the objective and fair scores provided by ASR technology can not only help learners address the problem without assessing their oral English by themselves (Randall et al., 2021), but also provide one-to-one feedback for learners, which greatly improves their learning efficiency (Dai and Wu, 2021; Bashori et al., 2022). Therefore, exploring the effectiveness of ASR technology has a certain research value for improving learners’ oral English level.

Some limitations of this paper should be pointed out. All participants were students of the same grade from the same university, which may lead to a low sample representation. Therefore, in future studies, researchers should explore whether ASR technology will have an impact on different language proficiency levels of learners. Additionally, the conclusions about the effectiveness of ASR in this paper are based on the self-feedback of learners. In order to further study the effectiveness of ASR technology for learners, researchers need to conduct in-depth research to understand how learners use ASR technology in the learning process. Meanwhile, it is also necessary to record the changes in learners’ language ability.

## Data availability statement

The original contributions presented in the study are included in the article/supplementary material, further inquiries can be directed to the corresponding author.

## Ethics statement

The studies involving human participants were reviewed and approved by Academic Committee of College of Foreign Languages, Bohai University, China. The patients/participants provided their written informed consent to participate in this study.

## Author contributions

JL and XL contributed equally to the conception of the idea, research design, implementing and analyzing the experimental results, writing the manuscript and so on. CY contributed to the data collection from the teaching experiment. All authors have read and approved the final version of the manuscript.

## Conflict of interest

The authors declare that the research was conducted in the absence of any commercial or financial relationships that could be construed as a potential conflict of interest.

## Publisher’s note

All claims expressed in this article are solely those of the authors and do not necessarily represent those of their affiliated organizations, or those of the publisher, the editors and the reviewers. Any product that may be evaluated in this article, or claim that may be made by its manufacturer, is not guaranteed or endorsed by the publisher.

## References

[ref1] Ahn T. Y. Lee S. M. (2016). User experience of a mobile speaking application with automatic speech recognition for EFL learning. Br. J. Educ. Technol. 47, 778–786. doi: 10.1111/bjet.12354

[ref2] Al-Sobhi B. M. S. Preece A. S. (2018). Teaching English speaking skills to the Arab students in the Saudi school in Kuala Lumpur: problems and solutions. Int. J. Educ. Lit. Stud. 6, 1–11. doi: 10.7575/aiac.ijels.v.6n.1p.1

[ref3] Arvanitis P. Krystalli P. (2021). Mobile assisted language learning (MALL): trends from 2010 to 2020 using text analysis techniques. Eur. J. Educ. 4, 13–22. doi: 10.26417/461iaw87u

[ref4] Bashori M. van Hout R. Strik H. Cucchiarini C. (2022). ‘Look, I can speak correctly’: learning vocabulary and pronunciation through websites equipped with automatic speech recognition technology. Comput. Assist. Lang. Learn., 1–29. doi: 10.1080/09588221.2022.2080230

[ref5] Bogach N. Boitsova E. Chernonog S. Lamtev A. Lesnichaya M. Lezhenin I.. (2021). Speech processing for language learning: a practical approach to computer-assisted pronunciation teaching. Electronics 10:235. doi: 10.3390/electronics10030235

[ref6] Bourdas D. I. Zacharakis E. D. (2020). Impact of COVID-19 lockdown on physical activity in a sample of Greek adults. Sports 8:139. doi: 10.3390/sports8100139 33096721 PMC7589063

[ref7] Bryman A. (2016). Social Research Methods. Oxford; New York, NY: Oxford University Press.

[ref8] Cavus N. (2016). Development of an intellegent mobile application for teaching English pronunciation. Procedia Comput. Sci. 102, 365–369. doi: 10.1016/j.procs.2016.09.413

[ref9] Cheng C. H. Chen C. H. (2022). Investigating the impacts of using a mobile interactive English learning system on the learning achievements and learning perceptions of student with different backgrounds. Comput. Assist. Lang. Learn. 35, 88–113. doi: 10.1080/09588221.2019.1671460

[ref10] Cicha K. Rizun M. Rutecka P. Strzelecki A. (2021). COVID-19 and higher education: first-year students’ expectations toward distance learning. Sustainability 13:1889. doi: 10.3390/su13041889

[ref11] Cohen L. Manion L. Morrison K. (2018). Research Methods in Education. 8th Edn. London; New York, NY: Routledge.

[ref12] Coskun A. (2016). Causes of the" I can understand English but I Can't speak" syndrome in Turkey. J. Engl. Lang. Teach. 6, 1–12.

[ref13] Dai Y. Wu Z. (2021). Mobile-assisted pronunciation learning with feedback from peers and/or automatic speech recognition: a mixed-methods study. Comput. Assist. Lang. Learn., 1–24. doi: 10.1080/09588221.2021.1952272

[ref14] Dalati S. Marx Gómez J. (2018). “Surveys and Questionnaires” in Modernizing the Academic Teaching and Research Environment. eds. Marx Gómez J. Mouselli S., S (Cham: Springer). 175–186.

[ref15] Egbert J. Akasha O. Huff L. Lee H. (2012). Moving forward: anecdotes and evidence guiding. Med. Appl. Intell. Data Anal. 1–15. doi: 10.4018/978-1-4666-1855-8.ch001

[ref16] Eisinga R. Grotenhuis M. T. Pelzer B. (2013). The reliability of a two-item scale: pearson, Cronbach, or spearman-Brown? Int. J. Public Health 58, 637–642. doi: 10.1007/s00038-012-0416-3 23089674

[ref17] Evers K. Chen S. (2020). Effects of an automatic speech recognition system with peer feedback on pronunciation instruction for adults. Comput. Assist. Lang. Learn., 1–21. doi: 10.1080/09588221.2020.1839504

[ref18] Gafni R. Achituv D. B. Rahmani G. (2017). Learning foreign languages using mobile applications. J. Inf. Technol. Educ. Res. 16:301. doi: 10.28945/3738

[ref19] Gehlbach H. Brinkworth M. E. (2011). Measure twice, cut down error: a process for enhancing the validity of survey scales. Rev. Gen. Psychol. 15, 380–387. doi: 10.1037/a0025704

[ref20] Golonka E. M. Bowles A. R. Frank V. M. Richardson D. L. Freynik S. (2014). Technologies for foreign language learning: a review of technology types and their effectiveness. Comput. Assist. Lang. Learn. 27, 70–105. doi: 10.1080/09588221.2012.700315

[ref21] Gonulal T. (2019). The use of Instagram as a mobile-assisted language learning tool. Contemp. Educ. Technol. 10, 309–323. doi: 10.30935/cet.590108

[ref22] Hashim H. Yunus M. M. Embi M. A. Ozir N. A. M. (2017). Mobile-assisted language learning (MALL) for ESL learners: a review of affordances and constraints. Sains Humanika. 45–50. doi: 10.11113/sh.v9n1-5.1175

[ref23] Kaendler C. Wiedmann M. Rummel N. Spada H. (2015). Teacher competencies for the implementation of collaborative learning in the classroom: a framework and research review. Educ. Psychol. Rev. 27, 505–536. doi: 10.1007/s10648-014-9288-9

[ref24] Kholis A. (2021). Elsa speak app: automatic speech recognition (ASR) for supplementing English pronunciation skills. Pedagogy J. Engl. Lang. Teach. 9, 01–14. doi: 10.32332/joelt.v9i1.2723

[ref25] Kondo M. Ishikawa Y. Smith C. Sakamoto K. Shimomura H. Wada N. (2012). Mobile assisted language learning in university EFL courses in Japan: developing attitudes and skills for self-regulated learning. ReCALL 24, 169–187. doi: 10.1017/s0958344012000055

[ref26] Kruk M. (2012). Using online resources in the development of learner autonomy and English pronunciation: the case of individual learners. J. Second Lang. Teach. Res. 1, 113–142.

[ref27] Lee J. Jang J. Plonsky L. (2015). The effectiveness of second language pronunciation instruction: a meta-analysis. Appl. Linguis. 36, 345–366. doi: 10.1093/applin/amu040

[ref28] Liakin D. Cardoso W. Liakina N. (2015). Learning L2 pronunciation with a mobile speech recognizer: French/y/. CALICO J. 32, 1–25. doi: 10.1558/cj.v32i1.25962

[ref29] Little D. G. (1991). Learner Autonomy: Definitions, Issues and Problems. Dublin: Authentik Language Learning Resources.

[ref30] Liu X. Xu M. Li M. Han M. Chen Z. Mo Y.. (2019). Improving English pronunciation via automatic speech recognition technology. Int. J. Innov. Learn. 25, 126–140. doi: 10.1504/ijil.2019.097674

[ref31] Mackey A. Gass S. M. (2021). Second Language Research: Methodology and Design. London; New York, NY: Routledge.

[ref32] McCrocklin S. M. (2016). Pronunciation learner autonomy: the potential of automatic speech recognition. System 57, 25–42. doi: 10.1016/j.system.2015.12.013

[ref33] Miangah T. M. Nezarat A. (2012). Mobile-assisted language learning. Int. J. Distrib. Parallel Syst. 3:309. doi: 10.5121/ijdps.2012.3126

[ref34] Miller R. L. Brewer J. D. (Eds.) (2003). The AZ of Social Research: A Dictionary of Key Social Science Research Concepts. London: Sage.

[ref35] Mortazavi M. Nasution M. K. Abdolahzadeh F. Behroozi M. Davarpanah A. (2021). Sustainable learning environment by mobile-assisted language learning approaches on the improvement of productive and receptive foreign language skills: a comparative study for Asian universities. Sustainability 13:6328. doi: 10.3390/su13116328

[ref36] Mroz A. (2018). Seeing how people hear you: French learners experiencing intelligibility through automatic speech recognition. Foreign Lang. Ann. 51, 617–637. doi: 10.1111/flan.12348

[ref37] Neri A. Cucchiarini C. Strik H. (2008). The effectiveness of computer-based speech corrective feedback for improving segmental quality in L2 Dutch. ReCALL 20, 225–243. doi: 10.1017/S0958344008000724

[ref38] Olson D. J. (2014). Benefits of visual feedback on segmental production in the L2 classroom. Lang. Learn. Technol. 18, 173–192.

[ref39] Pegrum M. (2019). Mobile Lenses on Learning. Springer, Singapore.

[ref40] Pourhosein Gilakjani A. Rahimy R. (2020). Using computer-assisted pronunciation teaching (CAPT) in English pronunciation instruction: a study on the impact and the Teacher’s role. Educ. Inf. Technol. 25, 1129–1159. doi: 10.1007/s10639-019-10009-1

[ref41] Rahimi M. Miri S. S. (2014). The impact of mobile dictionary use on language learning. Procedia Soc. Behav. Sci. 98, 1469–1474. doi: 10.1016/j.sbspro.2014.03.567

[ref42] Randall R. Hong Y. Nam H. (2021). The effect of real-time score feedback on L2 English learners’ pronunciation and motivation in an ASR-based CAPT system. Korean J. Appl. Linguist. 37, 7–50. doi: 10.17154/kjal.2021.12.37.4.7

[ref43] Rogerson-Revell P. M. (2021). Computer-assisted pronunciation training (CAPT): current issues and future directions. RELC J. 52, 189–205. doi: 10.1177/0033688220977406

[ref44] Saito K. Lyster R. (2012). Effects of form-focused instruction and corrective feedback on L2 pronunciation development of/ɹ/by Japanese learners of English. Lang. Learn. 62, 595–633. doi: 10.1111/j.1467-9922.2011.00639.x

[ref45] Sidgi L. F. S. Shaari A. J. (2017). The usefulness of automatic speech recognition (ASR) Eyespeak software in improving Iraqi EFL students’ pronunciation. Adv. Lang. Lit. Stud. 8, 221–226. doi: 10.7575/aiac.alls.v.8n.1p.221

[ref46] Stockwell G. (2022). Mobile Assisted Language Learning: Concepts, Contexts and Challenges. Cambridge; New York, NY: Cambridge University Press.

[ref47] Toland S. H. Mills D. J. Kohyama M. (2016). Enhancing Japanese university students’ English-language presentation skills with Mobile-video recordings. JALT CALL J. 12, 179–201.

[ref48] Van de Pol J. Volman M. Oort F. Beishuizen J. (2015). The effects of scaffolding in the classroom: support contingency and student independent working time in relation to student achievement, task effort and appreciation of support. Instr. Sci. 43, 615–641. doi: 10.1007/s11251-015-9351-z

[ref49] Wang Y. H. Young S. S. C. (2014). A study of the design and implementation of the ASR-based iCASL system with corrective feedback to facilitate English learning. J. Educ. Technol. Soc. 17, 219–233.

[ref50] Xodabande I. (2017). The effectiveness of social media network telegram in teaching English language pronunciation to Iranian EFL learners. Cogent Educ. 4:1347081. doi: 10.1080/2331186X.2017.1347081

[ref51] Zou B. Li H. Li J. (2018). Exploring a curriculum app and a social communication app for EFL learning. Comput. Assist. Lang. Learn. 31, 694–713. doi: 10.1080/09588221.2018.1438474

[ref52] Zou B. Yan X. (2014). Chinese students’ perceptions of using mobile devices for English learning. Int. J. Comput. Assist. Lang. Learn. Teach. 4, 20–33. doi: 10.4018/ijcallt.2014070102

